# District Medical Society—Organization Completed

**Published:** 1872-07-01

**Authors:** W. G. Cochran

**Affiliations:** Sec’y.


### DISTRICT MEDICAL SOCIETY—ORGANIZATION COMPLETED.

Pursuant to adjournment the physicians of Central Illinois met in Farmer City, July 2d, 1872, to complete the organization of "The Central Illinois Medical Society." Meeting called to order by Dr. C. T. Orner, Chairman.

Reports of committees being the first thing in order, the Committee on Constitution and By-laws, through their Chairman, Dr. John Wright, submitted a copy, which, after one or two amendments, was unanimously adopted.

Committee on Nominations, through their Chairman, Dr. Hill, reported:

*For President*—Dr. John Wright, Clinton.

*For First Vice President*—Dr. M. S. Brown, Urbana.

*For Second Vice President*—Dr. C. T. Orner, Saybrook.

*For Corresponding Secretary*—Dr. R. G. Laughlin, Bloomington.

*For Recording Secretary*—Dr. W. G. Cochran, Farmer City.

*For Treasurer*—Dr. H. C. Howard, Champaign.

*For Censors*—Dr. W. Hill, Bloomington; Dr. T. D. Fisher, Le Roy; Dr. J. T. Pearman, Champaign; Dr. C. Goodbrake, Clinton; Dr. J. S. Miller, Farmer City.

Report adopted.

Drs. Huddleston and Hill were appointed to conduct the President to his chair, when, by a few well timed remarks, he thanked the Society for the honor conferred upon him.

A motion that when we adjourn we do so to meet in Bloomington was carried unanimously.

Dr. R. G. Laughlin introduced the following resolution, which was adopted:

*Resolved*, That standing committees be appointed by the President on the subjects of Surgery, Practical Medicine, and Obstetrics, and that other Standing Committees be appointed at the option of the Society.

In accordance therewith the following committees were appointed:

*On Surgery*—Dr. C. Goodbrake, Clinton; Dr. W. Hill, Bloomington; Dr. J. T. Pearman, Champaign.

*On Practical Medicine*—Dr. R. G. Laughlin, Bloomington; Dr. J. D. Gardner, Mahomet; Dr. T. K. Edmiston, Clinton.

*On Obstetrics*—Dr. S. H. Birney, Urbana; Dr. T. D. Fisher, Le Roy; Dr. J. S. Miller, Farmer City.

*Committee of Arrangements*—Dr. R. G. Laughlin, Dr. W. Hill, and Dr. R. D. Bradley, all of Bloomington.

*Committee on Publication*—Dr. W. G. Cochran, Farmer City; Dr. S. H. Birney, Urbana; Dr. W. Hill, Bloomington.

Dr. Laughlin offered the following resolution, which was unanimously adopted:

*Resolved*, That the thanks of the Central Illinois Medical Society are hereby tendered to the Good Templars for the gratuitous use of their hall, and to the profession and citizens of Farmer City for their cordial hospitalities.

On motion of Dr. Hill, the Secretary was instructed to furnish copies of the proceedings for publication in the different county papers and the Chicago medical journals.

On motion, adjourned to meet in Bloomington, Illinois, on the second Tuesday in January, 1873.

Papers in the district, please copy.

W. G. Cochran, Sec'y.

